# (*Z*)-1-[(2*E*)-3,4-Diphenyl-2,3-di­hydro-1,3-thia­zol-2-yl­idene]-2-[1-(4-hy­droxy­phen­yl)ethyl­idene]hydrazinium bromide including an unknown solvate

**DOI:** 10.1107/S1600536814010125

**Published:** 2014-05-10

**Authors:** Joel T. Mague, Shaaban K. Mohamed, Mehmet Akkurt, Alaa A. Hassan, Mustafa R. Albayati

**Affiliations:** a Department of Chemistry, Tulane University, New Orleans, LA 70118, USA; b Chemistry and Environmental Division, Manchester Metropolitan University, Manchester M1 5GD, England; c Chemistry Department, Faculty of Science, Minia University, 61519 El-Minia, Egypt; d Department of Physics, Faculty of Sciences, Erciyes University, 38039 Kayseri, Turkey; e Kirkuk University, College of Science, Department of Chemistry, Kirkuk, Iraq

## Abstract

In the title compound, C_23_H_20_N_3_OS^+^·Br^−^, the di­hydro­thia­zole ring (r.m.s. deviation = 0.015 Å) is twisted with respect to each of the C- and N-bound phenyl rings and the hy­droxy­benzene ring, making dihedral angles of 76.0 (2), 71.2 (2) and 9.8 (2)°, respectively. In the crystal, inversion-related mol­ecules are linked by association of the bromide ions with the cations *via* N—H⋯Br and O—H⋯Br hydrogen-bonding inter­actions. These mol­ecules run in channels parallel to the *a* axis through face-to-face π–π stacking inter­actions between the hy­droxy­benzene rings [centroid–centroid distances = 3.785 (3) Å] which, in turn, are connected into layers parallel to (110) by weak C—H⋯π inter­actions. A small region of electron density well removed from the main mol­ecule and appearing disordered over a center of symmetry was removed with *PLATON* SQUEEZE [Spek (2009 ▶). *Acta Cryst.* D**65**, 148–15] following unsuccessful attempts to model it as plausible solvent molecule. The nature of the solvent was not known and hence, this is not taken into account when calculating *M* *_r_* and related data.

## Related literature   

For general medicinal and industrial applications of five-membered *S*,*N*-heterocycles thia­zolines, see: Abhinit *et al.* (2009 ▶). For chemical and diverse medicinal properties of thia­zoles, see: Sreedevi *et al.* (2013 ▶); Milne (2000 ▶); De Souza & De Almeida (2003 ▶); Lednicer *et al.* (1990 ▶); Rehman *et al.* (2005 ▶); Knadler *et al.* (1986 ▶). For a similar structure, see: Mague *et al.* (2014 ▶).
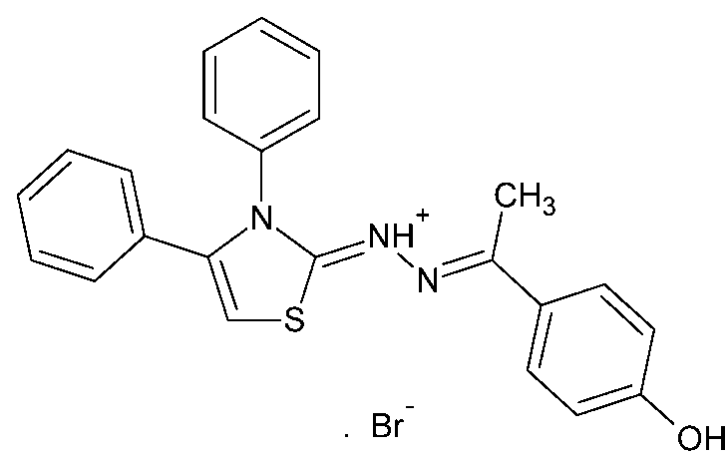


## Experimental   

### 

#### Crystal data  

C_23_H_20_N_3_OS^+^·Br^−^
*M* *_r_* = 466.39
Triclinic, 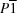

*a* = 7.5987 (12) Å
*b* = 12.3017 (19) Å
*c* = 13.786 (2) Å
α = 68.0760 (17)°
β = 88.1540 (18)°
γ = 72.6540 (18)°
*V* = 1136.6 (3) Å^3^
*Z* = 2
Mo *K*α radiation
μ = 1.92 mm^−1^
*T* = 220 K
0.17 × 0.17 × 0.12 mm

#### Data collection  

Bruker SMART APEX CCD diffractometer
Absorption correction: multi-scan (*SADABS*; Bruker, 2013 ▶) *T*_min_ = 0.56, *T*_max_ = 0.80
11044 measured reflections
5146 independent reflections
3398 reflections with *I* > 2σ(*I*)
*R*_int_ = 0.042

#### Refinement  

*R*[*F* ^2^ > 2σ(*F* ^2^)] = 0.058
*wR*(*F* ^2^) = 0.157
*S* = 1.01
5146 reflections
263 parameters
73 restraints
H-atom parameters constrained
Δρ_max_ = 1.09 e Å^−3^
Δρ_min_ = −0.48 e Å^−3^

### 

Data collection: *APEX2* (Bruker, 2013 ▶); cell refinement: *SAINT* (Bruker, 2013 ▶); data reduction: *SAINT*; program(s) used to solve structure: *SHELXS2013* (Sheldrick, 2008 ▶); program(s) used to refine structure: *SHELXL2013* (Sheldrick, 2008 ▶); molecular graphics: *ORTEP-3 for Windows* (Farrugia, 2012 ▶); software used to prepare material for publication: *WinGX* (Farrugia, 2012 ▶) and *PLATON* (Spek, 2009 ▶).

## Supplementary Material

Crystal structure: contains datablock(s) global, I. DOI: 10.1107/S1600536814010125/tk5311sup1.cif

Structure factors: contains datablock(s) I. DOI: 10.1107/S1600536814010125/tk5311Isup2.hkl

Supporting information file. DOI: 10.1107/S1600536814010125/tk5311Isup3.cml

CCDC reference: 1000895

Additional supporting information:  crystallographic information; 3D view; checkCIF report

## Figures and Tables

**Table 1:** Hydrogen-bond geometry (Å, °) *Cg*4 is the centroid of the C18–C23 benzene ring.

*D*—H⋯*A*	*D*—H	H⋯*A*	*D*⋯*A*	*D*—H⋯*A*
O1—H1*O*⋯Br1^i^	0.83	2.50	3.328 (4)	178
N2—H2⋯Br1^ii^	0.91	2.88	3.570 (3)	134
C5—H5⋯*Cg*4^iii^	0.94	2.76	3.610 (5)	152

Symmetry codes: (i) 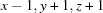; (ii) 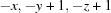; (iii) 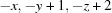.

**Fig. 1.**
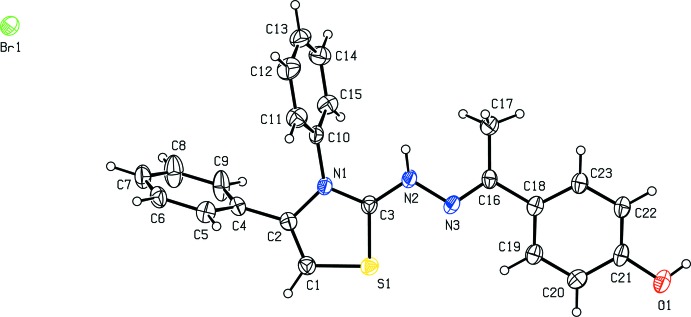
Perspective view of the asymmetric unit with 30% probability displacement ellipsoids.

**Fig. 2.**
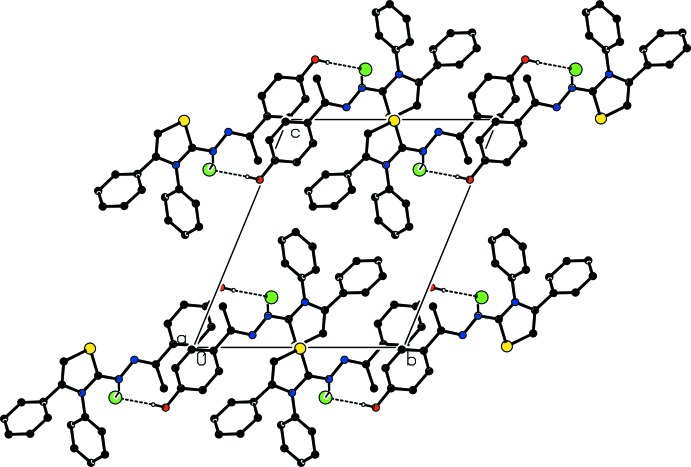
Packing viewed down the a axis and showing O—H···Br interactions.

## supplementary crystallographic information

### 1. Structural commentary

Synthesis of five-membered *S*,*N*-heterocycles thia­zolines has received intensive inter­est from chemists and biologists due to their wide spectrum of medicinal and industrial applications (Abhinit *et al.*, 2009). Thia­zoles are stable and non-carcinogenic aromatic compounds with relatively small size (Sreedevi *et al.*, 2013). Many biologically active products, such as Bleomycin and Tiazofurin (anti­neoplastic agents) (Milne, 2000), Ritonavir (anti-HIV drug) (De Souza & De Almeida, 2003), Fanetizole and Meloxicam (anti-inﬂammatory agents) (Lednicer *et al.*, 1990; Rehman *et al.*, 2005), Nizatidine (anti­ulcer agent) (Knadler *et al.*, 1986) and penicillin (anti­biotic) are some examples of thia­zole-bearing compounds.

In the title molecule (I), shown in Fig. 1, the 5-membered heterocycle (S1/N1/C1–C3) is planar to within 0.013 (3) Å for N1 and the phenyl rings C4–C9 and C10–C15 make dihedral angles with it of 76.0 (2)° and 71.2 (2)°, respectively. The dihedral angle between the ring C18–C23 and the heterocycle (S1/N1/C1–C3) is 9.8 (2)°. The N1–C3–N2–N3, C3–N2–N3–C18, N2–N3–C16–C18 torsion angles are 173.6 (4), 166.3 (4) and -177.1 (3)°, respectively. All bond lengths and bond angles in (I) are normal and are comparable with those found in a similar structure (Mague *et al.*, 2014).

In the crystal, inversion-related molecules are linked by association of the bromide ions with the cations *via* N—H···Br and O—H···Br hydrogen bonding inter­actions (Table 1, Fig. 2). These molecules run in channels parallel to the *a* axis through the face-to-face π—π stacking inter­actions [centroid-to-centroid distances = 3.785 (3) Å] between the hydroxyl­benzene rings: these are connected into layers parallel to (110) by weak C5—H5···Cg(C18–C23) inter­actions, Table 1.

### 2. Synthesis and crystallization

The title compound was prepared according to our reported method (Mague *et al*., 2014). The crude product has been crystallized from ethanol to afford colorless crystals suitable for X-ray diffraction (m.p.: 533 – 535 K).

### 3. Refinement

All H atoms were fixed geometrically and treated as riding with C—H= 0.94–0.97 Å, N–H = 0.91 Å and O–H = 0.83 Å, and with *U*_iso_(H)= 1.2*U*_eq_(C,*N*) and 1.5 *U*_eq_(O). A small region of electron density well–removed from the main molecule and appearing disordered over a center of symmetry was removed with *PLATON SQUEEZE* following unsuccessful attempts to model it as plausible lattice solvent (Spek, 2009).

### Crystal data

**Table:**

C_23_H_20_N_3_OS^+^·Br^−^	*Z* = 2
*M**_r_* = 466.39	*F*(000) = 476
Triclinic, *P*1	*D*_x_ = 1.363 Mg m^−^^3^
Hall symbol: -P 1	Mo *K*α radiation, λ = 0.71073 Å
*a* = 7.5987 (12) Å	Cell parameters from 4151 reflections
*b* = 12.3017 (19) Å	θ = 2.8–26.7°
*c* = 13.786 (2) Å	µ = 1.92 mm^−^^1^
α = 68.0760 (17)°	*T* = 220 K
β = 88.1540 (18)°	Block, colourless
γ = 72.6540 (18)°	0.17 × 0.17 × 0.12 mm
*V* = 1136.6 (3) Å^3^	

### Data collection

**Table:**

Bruker SMART APEX CCD diffractometer	5146 independent reflections
Radiation source: fine-focus sealed tube	3398 reflections with *I* > 2σ(*I*)
Graphite monochromator	*R*_int_ = 0.042
Detector resolution: 8.3660 pixels mm^-1^	θ_max_ = 27.5°, θ_min_ = 1.9°
φ and ω scans	*h* = −9→9
Absorption correction: multi-scan (*SADABS*; Bruker, 2013)	*k* = −15→15
*T*_min_ = 0.56, *T*_max_ = 0.80	*l* = −17→17
11044 measured reflections	

### Refinement

**Table:**

Refinement on *F*^2^	73 restraints
Least-squares matrix: full	Hydrogen site location: mixed
*R*[*F*^2^ > 2σ(*F*^2^)] = 0.058	H-atom parameters constrained
*wR*(*F*^2^) = 0.157	*w* = 1/[σ^2^(*F*_o_^2^) + (0.0867*P*)^2^] where *P* = (*F*_o_^2^ + 2*F*_c_^2^)/3
*S* = 1.01	(Δ/σ)_max_ < 0.001
5146 reflections	Δρ_max_ = 1.09 e Å^−^^3^
263 parameters	Δρ_min_ = −0.48 e Å^−^^3^

### Special details

**Table:**

Geometry. Bond distances, angles *etc*. have been calculated using the rounded fractional coordinates. All su's are estimated from the variances of the (full) variance-covariance matrix. The cell e.s.d.'s are taken into account in the estimation of distances, angles and torsion angles
Refinement. Refinement of *F*^2^ against ALL reflections. The weighted *R*-factor *wR* and goodness of fit *S* are based on *F*^2^, conventional *R*-factors *R* are based on *F*, with *F* set to zero for negative *F*^2^. The threshold expression of *F*^2^ > σ(*F*^2^) is used only for calculating *R*-factors(gt) *etc*. and is not relevant to the choice of reflections for refinement. *R*-factors based on *F*^2^ are statistically about twice as large as those based on *F*, and *R*- factors based on ALL data will be even larger. H-atoms attached to carbon were placed in calculated positions (C—H = 0.95 - 0.98 Å) while those attached to nitrogen were placed in locations derived from a difference map and their coordinates adjusted to give N—H = 0.91 Å. That attached to oxygen was placed in an idealized position and the C—C—O—H torsion angle refined (AFIX 147)·All were included as riding contributions with isotropic displacement parameters 1.2 - 1.5 times those of the attached atoms. A small region of electron density well removed from the main molecule and appearing disordered over a center of symmetry was removed with *PLATON SQUEEZE* following unsuccessful attempts to model it as plausible lattice solvent (Spek, 2014).

### Fractional atomic coordinates and isotropic or equivalent isotropic displacement parameters (Å^2^)

**Table:**

	*x*	*y*	*z*	*U*_iso_*/*U*_eq_	
S1	0.24831 (13)	0.51039 (10)	0.99502 (8)	0.0418 (3)	
O1	−0.1920 (4)	1.0140 (3)	1.2638 (2)	0.0599 (11)	
N1	0.1776 (4)	0.5582 (3)	0.8008 (2)	0.0376 (10)	
N2	−0.0083 (4)	0.7072 (3)	0.8605 (2)	0.0427 (11)	
N3	−0.0284 (5)	0.7434 (3)	0.9459 (2)	0.0434 (11)	
C1	0.3784 (5)	0.4108 (4)	0.9384 (3)	0.0433 (12)	
C2	0.3282 (5)	0.4476 (4)	0.8368 (3)	0.0399 (12)	
C3	0.1253 (5)	0.6024 (4)	0.8761 (3)	0.0393 (12)	
C4	0.4141 (5)	0.3930 (4)	0.7614 (3)	0.0398 (12)	
C5	0.3820 (6)	0.2897 (4)	0.7589 (3)	0.0480 (16)	
C6	0.4610 (6)	0.2396 (4)	0.6870 (4)	0.0510 (16)	
C7	0.5711 (7)	0.2920 (5)	0.6183 (4)	0.0604 (17)	
C8	0.6055 (8)	0.3930 (5)	0.6209 (5)	0.085 (3)	
C9	0.5280 (7)	0.4447 (5)	0.6915 (4)	0.070 (2)	
C10	0.0904 (5)	0.6216 (4)	0.6942 (3)	0.0389 (12)	
C11	−0.0203 (6)	0.5700 (4)	0.6591 (4)	0.0543 (17)	
C12	−0.1016 (7)	0.6310 (5)	0.5574 (4)	0.0689 (19)	
C13	−0.0764 (6)	0.7396 (5)	0.4938 (4)	0.0608 (18)	
C14	0.0333 (6)	0.7896 (5)	0.5299 (4)	0.0576 (17)	
C15	0.1188 (6)	0.7291 (4)	0.6313 (3)	0.0495 (14)	
C16	−0.1282 (5)	0.8544 (4)	0.9258 (3)	0.0380 (12)	
C17	−0.2232 (6)	0.9401 (4)	0.8202 (3)	0.0478 (14)	
C18	−0.1445 (5)	0.8977 (4)	1.0145 (3)	0.0388 (12)	
C19	−0.0230 (6)	0.8316 (4)	1.1041 (3)	0.0443 (12)	
C20	−0.0409 (6)	0.8719 (4)	1.1875 (3)	0.0480 (16)	
C21	−0.1813 (6)	0.9791 (4)	1.1794 (3)	0.0446 (14)	
C22	−0.2998 (6)	1.0452 (4)	1.0906 (3)	0.0470 (14)	
C23	−0.2808 (6)	1.0045 (4)	1.0093 (3)	0.0451 (14)	
Br1	0.46263 (6)	0.27288 (4)	0.21975 (3)	0.0504 (2)	
H1	0.47420	0.33840	0.97570	0.0520*	
H1O	−0.27870	1.07870	1.25100	0.0900*	
H2	−0.09160	0.73360	0.80430	0.0510*	
H5	0.30560	0.25280	0.80650	0.0570*	
H6	0.43800	0.16900	0.68600	0.0620*	
H7	0.62360	0.25870	0.56900	0.0730*	
H8	0.68370	0.42830	0.57370	0.1020*	
H9	0.55280	0.51490	0.69200	0.0830*	
H11	−0.03950	0.49580	0.70320	0.0650*	
H12	−0.17560	0.59700	0.53150	0.0830*	
H13	−0.13440	0.78040	0.42500	0.0730*	
H14	0.05050	0.86450	0.48600	0.0690*	
H15	0.19550	0.76220	0.65630	0.0590*	
H17A	−0.26820	1.02330	0.81860	0.0720*	
H17B	−0.32660	0.91560	0.80600	0.0720*	
H17C	−0.13670	0.93690	0.76730	0.0720*	
H19	0.07160	0.75970	1.10900	0.0530*	
H20	0.04110	0.82700	1.24860	0.0580*	
H22	−0.39340	1.11780	1.08500	0.0570*	
H23	−0.36270	1.05060	0.94830	0.0540*	

### Atomic displacement parameters (Å^2^)

**Table:**

	*U*^11^	*U*^22^	*U*^33^	*U*^12^	*U*^13^	*U*^23^
S1	0.0397 (6)	0.0478 (6)	0.0404 (5)	−0.0129 (5)	0.0012 (4)	−0.0197 (5)
O1	0.068 (2)	0.069 (2)	0.0568 (19)	−0.0195 (17)	0.0135 (15)	−0.0413 (17)
N1	0.0332 (17)	0.0438 (19)	0.0401 (17)	−0.0070 (14)	0.0016 (13)	−0.0244 (15)
N2	0.0413 (19)	0.050 (2)	0.0405 (18)	−0.0047 (15)	−0.0006 (14)	−0.0282 (16)
N3	0.050 (2)	0.050 (2)	0.0363 (17)	−0.0139 (17)	0.0061 (14)	−0.0244 (16)
C1	0.038 (2)	0.045 (2)	0.047 (2)	−0.0093 (18)	0.0020 (17)	−0.0202 (19)
C2	0.036 (2)	0.040 (2)	0.046 (2)	−0.0114 (17)	0.0024 (16)	−0.0192 (18)
C3	0.036 (2)	0.044 (2)	0.044 (2)	−0.0112 (17)	0.0021 (16)	−0.0243 (18)
C4	0.035 (2)	0.036 (2)	0.049 (2)	−0.0077 (17)	0.0045 (16)	−0.0193 (18)
C5	0.047 (2)	0.052 (3)	0.057 (3)	−0.026 (2)	0.0103 (19)	−0.026 (2)
C6	0.049 (3)	0.045 (2)	0.067 (3)	−0.009 (2)	−0.001 (2)	−0.034 (2)
C7	0.059 (3)	0.058 (3)	0.068 (3)	−0.008 (2)	0.018 (2)	−0.037 (3)
C8	0.092 (4)	0.073 (4)	0.114 (5)	−0.043 (3)	0.065 (4)	−0.054 (4)
C9	0.076 (4)	0.059 (3)	0.104 (4)	−0.038 (3)	0.050 (3)	−0.054 (3)
C10	0.033 (2)	0.046 (2)	0.042 (2)	−0.0056 (17)	0.0046 (16)	−0.0266 (18)
C11	0.052 (3)	0.060 (3)	0.055 (3)	−0.020 (2)	0.000 (2)	−0.024 (2)
C12	0.060 (3)	0.090 (4)	0.070 (3)	−0.024 (3)	−0.013 (2)	−0.043 (3)
C13	0.045 (3)	0.085 (4)	0.044 (2)	−0.006 (2)	−0.0011 (19)	−0.026 (2)
C14	0.049 (3)	0.062 (3)	0.050 (3)	−0.011 (2)	0.003 (2)	−0.013 (2)
C15	0.044 (2)	0.060 (3)	0.047 (2)	−0.020 (2)	0.0041 (18)	−0.020 (2)
C16	0.031 (2)	0.043 (2)	0.045 (2)	−0.0111 (17)	0.0097 (16)	−0.0230 (18)
C17	0.051 (3)	0.049 (2)	0.048 (2)	−0.016 (2)	0.0018 (18)	−0.023 (2)
C18	0.038 (2)	0.046 (2)	0.043 (2)	−0.0223 (18)	0.0144 (16)	−0.0223 (18)
C19	0.047 (2)	0.048 (2)	0.048 (2)	−0.0208 (19)	0.0112 (18)	−0.025 (2)
C20	0.050 (3)	0.055 (3)	0.044 (2)	−0.022 (2)	0.0069 (18)	−0.020 (2)
C21	0.049 (2)	0.053 (3)	0.050 (2)	−0.026 (2)	0.0232 (19)	−0.033 (2)
C22	0.043 (2)	0.055 (3)	0.052 (2)	−0.015 (2)	0.0135 (18)	−0.031 (2)
C23	0.041 (2)	0.052 (3)	0.051 (2)	−0.0170 (19)	0.0138 (18)	−0.028 (2)
Br1	0.0562 (3)	0.0514 (3)	0.0488 (3)	−0.0152 (2)	−0.0025 (2)	−0.0252 (2)

### Geometric parameters (Å, º)

**Table:**

S1—C1	1.741 (5)	C16—C18	1.494 (6)
S1—C3	1.710 (4)	C18—C23	1.388 (7)
O1—C21	1.375 (5)	C18—C19	1.384 (6)
O1—H1O	0.8300	C19—C20	1.401 (6)
N1—C3	1.341 (5)	C20—C21	1.396 (7)
N1—C10	1.451 (5)	C21—C22	1.365 (6)
N1—C2	1.418 (6)	C22—C23	1.376 (6)
N2—C3	1.329 (6)	C1—H1	0.9400
N2—N3	1.395 (4)	C5—H5	0.9400
N3—C16	1.277 (6)	C6—H6	0.9400
N2—H2	0.9100	C7—H7	0.9400
C1—C2	1.331 (5)	C8—H8	0.9400
C2—C4	1.474 (6)	C9—H9	0.9400
C4—C9	1.378 (7)	C11—H11	0.9400
C4—C5	1.376 (7)	C12—H12	0.9400
C5—C6	1.386 (7)	C13—H13	0.9400
C6—C7	1.352 (7)	C14—H14	0.9400
C7—C8	1.357 (9)	C15—H15	0.9400
C8—C9	1.379 (9)	C17—H17A	0.9700
C10—C11	1.385 (7)	C17—H17B	0.9700
C10—C15	1.360 (6)	C17—H17C	0.9700
C11—C12	1.380 (7)	C19—H19	0.9400
C12—C13	1.363 (8)	C20—H20	0.9400
C13—C14	1.372 (8)	C22—H22	0.9400
C14—C15	1.387 (6)	C23—H23	0.9400
C16—C17	1.490 (6)		
			
C1—S1—C3	89.3 (2)	O1—C21—C20	116.9 (4)
C21—O1—H1O	109.00	C20—C21—C22	120.5 (4)
C2—N1—C10	125.3 (3)	C21—C22—C23	119.3 (5)
C3—N1—C10	122.2 (4)	C18—C23—C22	122.1 (4)
C2—N1—C3	112.5 (3)	S1—C1—H1	124.00
N3—N2—C3	114.7 (3)	C2—C1—H1	123.00
N2—N3—C16	115.3 (3)	C4—C5—H5	120.00
C3—N2—H2	115.00	C6—C5—H5	120.00
N3—N2—H2	128.00	C5—C6—H6	120.00
S1—C1—C2	113.1 (4)	C7—C6—H6	120.00
N1—C2—C1	111.8 (4)	C6—C7—H7	120.00
N1—C2—C4	119.4 (3)	C8—C7—H7	120.00
C1—C2—C4	128.7 (4)	C7—C8—H8	119.00
S1—C3—N1	113.3 (3)	C9—C8—H8	119.00
N1—C3—N2	123.7 (3)	C4—C9—H9	120.00
S1—C3—N2	123.0 (3)	C8—C9—H9	120.00
C5—C4—C9	118.5 (4)	C10—C11—H11	121.00
C2—C4—C5	120.9 (4)	C12—C11—H11	121.00
C2—C4—C9	120.6 (5)	C11—C12—H12	119.00
C4—C5—C6	120.7 (4)	C13—C12—H12	119.00
C5—C6—C7	120.1 (5)	C12—C13—H13	120.00
C6—C7—C8	119.7 (5)	C14—C13—H13	120.00
C7—C8—C9	121.2 (6)	C13—C14—H14	120.00
C4—C9—C8	119.8 (6)	C15—C14—H14	120.00
C11—C10—C15	121.6 (4)	C10—C15—H15	120.00
N1—C10—C11	118.6 (4)	C14—C15—H15	120.00
N1—C10—C15	119.8 (4)	C16—C17—H17A	109.00
C10—C11—C12	117.9 (5)	C16—C17—H17B	109.00
C11—C12—C13	121.2 (5)	C16—C17—H17C	109.00
C12—C13—C14	120.1 (5)	H17A—C17—H17B	110.00
C13—C14—C15	119.8 (5)	H17A—C17—H17C	110.00
C10—C15—C14	119.4 (4)	H17B—C17—H17C	109.00
C17—C16—C18	120.0 (4)	C18—C19—H19	120.00
N3—C16—C18	116.5 (3)	C20—C19—H19	120.00
N3—C16—C17	123.5 (4)	C19—C20—H20	120.00
C16—C18—C19	120.4 (4)	C21—C20—H20	120.00
C16—C18—C23	121.1 (4)	C21—C22—H22	120.00
C19—C18—C23	118.5 (4)	C23—C22—H22	120.00
C18—C19—C20	120.0 (4)	C18—C23—H23	119.00
C19—C20—C21	119.6 (4)	C22—C23—H23	119.00
O1—C21—C22	122.6 (4)		
			
C3—S1—C1—C2	0.0 (4)	C2—C4—C9—C8	−179.3 (5)
C1—S1—C3—N1	1.4 (3)	C5—C4—C9—C8	0.4 (7)
C1—S1—C3—N2	−178.5 (4)	C4—C5—C6—C7	0.1 (7)
C3—N1—C2—C1	2.3 (5)	C5—C6—C7—C8	0.7 (8)
C3—N1—C2—C4	−174.3 (4)	C6—C7—C8—C9	−1.0 (9)
C10—N1—C2—C1	−179.8 (4)	C7—C8—C9—C4	0.4 (9)
C10—N1—C2—C4	3.6 (6)	N1—C10—C11—C12	−179.7 (4)
C2—N1—C3—S1	−2.4 (4)	C15—C10—C11—C12	0.2 (7)
C2—N1—C3—N2	177.5 (4)	N1—C10—C15—C14	−179.4 (4)
C10—N1—C3—S1	179.6 (3)	C11—C10—C15—C14	0.8 (7)
C10—N1—C3—N2	−0.5 (6)	C10—C11—C12—C13	−1.0 (8)
C2—N1—C10—C11	72.5 (5)	C11—C12—C13—C14	0.9 (9)
C2—N1—C10—C15	−107.4 (5)	C12—C13—C14—C15	0.1 (8)
C3—N1—C10—C11	−109.8 (5)	C13—C14—C15—C10	−0.9 (8)
C3—N1—C10—C15	70.3 (5)	N3—C16—C18—C19	15.2 (6)
C3—N2—N3—C16	166.3 (4)	N3—C16—C18—C23	−164.8 (4)
N3—N2—C3—S1	6.3 (5)	C17—C16—C18—C19	−163.8 (4)
N3—N2—C3—N1	−173.6 (4)	C17—C16—C18—C23	16.2 (6)
N2—N3—C16—C17	1.9 (6)	C16—C18—C19—C20	−179.1 (4)
N2—N3—C16—C18	−177.1 (3)	C23—C18—C19—C20	0.9 (7)
S1—C1—C2—N1	−1.3 (5)	C16—C18—C23—C22	179.2 (4)
S1—C1—C2—C4	175.0 (4)	C19—C18—C23—C22	−0.9 (7)
N1—C2—C4—C5	−105.2 (5)	C18—C19—C20—C21	−0.2 (7)
N1—C2—C4—C9	74.5 (6)	C19—C20—C21—O1	180.0 (4)
C1—C2—C4—C5	78.8 (6)	C19—C20—C21—C22	−0.6 (7)
C1—C2—C4—C9	−101.5 (6)	O1—C21—C22—C23	−179.9 (4)
C2—C4—C5—C6	179.1 (4)	C20—C21—C22—C23	0.6 (7)
C9—C4—C5—C6	−0.6 (7)	C21—C22—C23—C18	0.1 (7)

### Hydrogen-bond geometry (Å, º)

Cg4 is the centroid of the C18–C23 benzene ring.

**Table:**

*D*—H···*A*	*D*—H	H···*A*	*D*···*A*	*D*—H···*A*
O1—H1*O*···Br1^i^	0.83	2.50	3.328 (4)	178
N2—H2···Br1^ii^	0.91	2.88	3.570 (3)	134
C5—H5···*Cg*4^iii^	0.94	2.76	3.610 (5)	152

Symmetry codes: (i) *x*−1, *y*+1, *z*+1; (ii) −*x*, −*y*+1, −*z*+1; (iii) −*x*, −*y*+1, −*z*+2.
